# Treatment and control of low-density lipoprotein for primary prevention in patients in Wales with and without depression: a study of whole-population electronic health records

**DOI:** 10.1136/openhrt-2025-003800

**Published:** 2026-06-22

**Authors:** Carla White, Richard Summers, Ann John, David PJ Osborn, Keith Lloyd, Ashley Akbari, Michael B Gravenor, Julian P Halcox, Elizabeth A Ellins

**Affiliations:** 1 Swansea University Medical School, Swansea University, Swansea, Wales, UK; 2 Division of Psychiatry, University College London, London, UK

**Keywords:** DEPRESSION, RISK FACTORS, LIPIDS

## Abstract

**Aim:**

This study investigated the influence of depression status on lipid lowering therapy (LLT) prescribing and achievement of guideline targets for low density lipoprotein cholesterol (LDL-C) levels in patients after first documentation of a high risk of developing atherosclerotic cardiovascular disease (ASCVD). Associations with sex, socioeconomic status and location of residence on these relationships were also explored.

**Methods:**

A retrospective observational cohort study (2010–2019) using individual-level linked, anonymised, routinely collected electronic health record data sources. Patients with/without depression and documentation of a high global QRISK risk score (HQR) were included. Outcome variables were LLT prescription within 6 months of HQR documentation and recording of LDL-C level within European Society of Cardiology/European Atherosclerosis Society (ESC/EAS) targets and achievement of >40% reduction in LDL-C according to National Institute for Health and Care Excellence guidance within 1 year of HQR. Logistic regression analysis explored the association between depression and outcome variables adjusting for sex, age group, deprivation, location of residence and other risk factors.

**Results:**

QRISK was documented in 284 859 (12.8%) patients. In the 103 340 HQR patients, depression (identified pre-HQR or post-HQR documentation) was associated with a higher likelihood of LLT prescription (pre-OR 1.15, 95% CI 1.08 to 1.23; post-OR 1.39, 95% CI 1.19 to 1.64). Depression was not associated with achievement of LDL-C control as per EAS/ESC guidelines (<2.6 mmol/L; pre-OR 1.10, 95% CI 1.00 to 1.23; post-OR 1.00, 95% CI 0.79 to 1.28). Depression pre-HQR was associated with achievement of a >40% reduction in LDL-C (pre-OR 1.16, 95% CI 1.02 to 1.32; post-OR 0.94, 95% CI 0.70 to 1.25)

**Conclusion:**

Only a small proportion of patients had a documented QRISK score in their record. While high ASCVD risk patients with depression were more likely to be prescribed LLT, this was not necessarily associated with better LDL-C control.

**WHAT IS ALREADY KNOWN ON THIS TOPIC**

**WHAT THIS STUDY ADDS**

- Patients with depression diagnosed before or after documentation of a high cardiovascular risk score (HQR) in the primary care record were more likely to be prescribed lipid lowering therapy than non-depressed patients after documentation of HQR. However, this did not necessarily result in greater achievement of guideline targets for lipid levels. Documentation of 10-year risk was infrequent and follow-up monitoring of lipid levels in those with documented HQR was poor, irrespective of depression status.

**HOW THIS STUDY MIGHT AFFECT RESEARCH, PRACTICE OR POLICY**

- Documentation of cardiovascular risk status and lipid assessment needs to be improved across the whole population for primary prevention of cardiovascular disease. Further work is required to investigate why patients with depression are no more likely to achieve guideline lipid targets despite greater prescribing of lipid lowering therapy.

## Introduction

 Optimising lipid levels is a cornerstone in the prevention of atherosclerotic cardiovascular disease (ASCVD), reducing the risk of a first or subsequent cardiovascular event. Lowering low density lipoprotein cholesterol (LDL-C) levels by 1 mmol/L has been shown to reduce the risk of major vascular events by just over one fifth, demonstrating the benefits of lipid lowering therapy (LLT) for patients with ASCVD and at increased risk of CVD events.^1^ Effective management of lipids and other major cardiovascular risk factors therefore is of significant benefit to such patients. How well this is carried out in routine care is not clear, particularly in patients who may have additional risk from other non-traditional risk factors for ASCVD, such as depression.^2^ A previous study by our group showed that coronary artery disease patients with depression have their lipids less well monitored and controlled following percutaneous coronary intervention than those without depression, suggesting they may be less likely to adhere to preventative medication and consequently not reach targets for lipid levels.^3 4^ However, whether patients with depression in the general population who are free from but at high risk of developing ASCVD are prescribed recommended LLT and achieve guideline-recommended lipid targets is not known.

Aside from the additional cardiovascular risk of depression, other factors may impact risk factor management, such as sex, socioeconomic status and where individuals live. Females are more likely to develop depression, and studies have shown that women are less likely than men to be prescribed LLT and achieve guideline targets.^5^,^7^ However, these studies have not looked specifically at the additional impact of depression in the primary prevention setting. Our group has previously shown that females with co-morbid depression were the least likely to have lipid testing and achieve LDL-C guideline targets after percutaneous coronary intervention than non-depressed females and males, both with and without depression.^3^ Socioeconomic factors and location of residence (eg, urban vs rural) may also influence quality of care, but studies examining this issue are inconclusive and vary according to study population.^8^,^14^ Thus, there is a pressing need to determine the demographic and clinical factors that might potentially influence lipid management in patients at high risk of ASCVD.

Therefore, we investigated the influence of depression status on the likelihood of receiving guideline-recommended LLT and documentation of achievement of guideline targets for LDL-C levels in primary prevention patients at high risk of ASCVD. Additionally, we explored the associations with sex, socioeconomic status and living in an urban or rural environment on these relationships.

## Methods

### Cohort

Access to data and linkage was through the privacy-protecting trusted research environment, the Secure Anonymised Information Linkage (SAIL) Databank.^15 16^ Patients for this analysis came from a retrospective observational cohort formed from individual-level linked anonymised electronic health record (EHR) data sources for patients who were free from ASCVD, depression or severe mental illness, aged 18 years or over and had at least 1 year of data within SAIL prior to entry into the cohort. Patients entered the original cohort on 1 January 2010 or the date of achievement of the inclusion criteria. Patients remained in the cohort until 31 December 2019, death or on leaving a SAIL providing general practice.^17^ The code used to generate the cohort is also available (https://github.com/r-sum-1/SAIL0800-CVD-Depression.git).

### Study population: patients at high cardiovascular risk

Patients from the original cohort with a documented high cardiovascular risk score QRISK (HQR) by their general practitioner (GP) and at least 90 days follow-up were included in this evaluation. QRISK scores, defined as ≥20% 10-year risk prior to 2015 and ≥10% from 2015 onwards, defined patients as being at high risk of ASCVD. QRISK is the risk calculator recommended by the National Institute for Health and Care Excellence (NICE) guidelines for GPs to identify patients at risk of ASCVD. The change in HQR definition from 2015 reflects the change in the NICE clinical guideline 10year risk threshold to define high risk for initiation of LLT from ≥20% to ≥10% (NICE CG181 replaced by NG238).^18^

The date of first HQR documentation in the EHR was taken as the index date for the outcome analysis for all patients.

### Exposure: depression characterisation

Diagnostic (Read) codes based on previous work were used to identify patients with a record of any of the following: diagnosis of depression or mixed anxiety and depression, anxiety, severe mental illness (including bipolar disorder, schizophrenia and other psychotic illnesses), depressive symptoms, anxiety symptoms and prescriptions for antidepressants or anxiolytics from the Welsh Longitudinal General Practice (WLGP) data.^19^ Patients who met the following criteria, diagnosis of depression or mixed anxiety and depression in their medical history or a record of depressive symptoms together with a prescription of antidepressants within 6 months of record of the depressive symptoms, were categorised as having depression during the study period.^20 21^ Inclusion of depressive symptoms treated with antidepressants within the depression categorisation reflects changes within GP diagnostic coding behaviour and has been validated by John *et al*.^19^ Patients prescribed antidepressants but without a record of depression diagnosis or symptoms were not categorised as depressed, given they may have been prescribed antidepressants for other clinical indications, for example, pain, anxiety or obsessive compulsive disorder. All patients entered the cohort at HQR documentation. Patients coded with depression before the first documentation of HQR (index date) were categorised as depressed pre-HQR, while those coded with depression within 1 year of first HQR score documentation were termed depressed post-HQR diagnosis. Patients who did not receive a qualifying depression code during the study period were categorised as non-depressed.

### Outcomes

#### Lipid treatment within 6 months of first documented high QRISK score

Prescriptions for LLT, including statins (high and normal intensity), ezetimibe and fibrates within 6 months of HQR documentation were identified within WLGP data. LLT initiation was taken as the date of first prescription. Patients prescribed LLT before first documentation of a HQR score were excluded from the analysis.

### Achievement of guideline-recommended LDL-C levels

The lowest LDL-C (estimated from the Friedewald formula), within 1 year post first HQR documentation was recorded from the WLGP and Welsh Results Reporting Service data.^22^ The number (and proportion) of patients achieving the European Society of Cardiology/European Atherosclerosis Society (ESC/EAS) 2016 lipid guidelines targets for LDL-C of <2.6 mmol/L were identified.^23^ The 2016 guideline targets were used as these were similar to previous versions of the guidelines and covered the period of the study. In addition, patients who also had a LDL-C level recorded in the year prior to index date of HQR documentation were identified to assess achievement of ≥40% reduction in LDL-C as per the target in the NICE guidelines.^18^ While the NICE guidelines recommended assessing non-high-density lipoprotein cholesterol levels, this was rarely documented in the primary care record during the study period, so LDL-C reduction was used as our primary outcome measure for lipid control.

### Covariates

Presence of hypertension, ischaemic heart disease, chronic kidney disease (CKD) stage 4+ and chronic liver disease was identified from primary care data along with patients’ age. The Welsh Demographic Service Dataset was used to capture patient’s sex and to link other variables such as the lower-layer super output area V.2001 of residence and from this linkage to the area-based deprivation measure Welsh Index of Multiple Deprivation 2011, an indicator of socioeconomic status and to provide location (rural and urban).

### Statistical analysis

Comparisons between the non-depressed, depression pre-HQR documentation and depression post-HQR documentation were carried out using analysis of variance (ANOVA) or χ^2^ as appropriate.

Logistic regression was used to investigate whether depression status was associated with the main outcomes of LLT within 6 months of index date and achievement of LDL-C target within 1 year of HQR documentation. Models were adjusted for sex, age group (18–39 years, 40–59 years, 60–74 years and 75+ years), deprivation, location of residence, smoking status, history of diabetes, CKD stage 4+, liver disease and hypertension. History was taken as prior to date of HQR documentation. LLT status was also included in the control outcome. For all analyses, interactions between depression*sex, depression*age group, depression*deprivation and depression*location of residence were explored for each outcome. Analyses were carried out using R V.2024.24.0.

## Results

**Figure 1:**
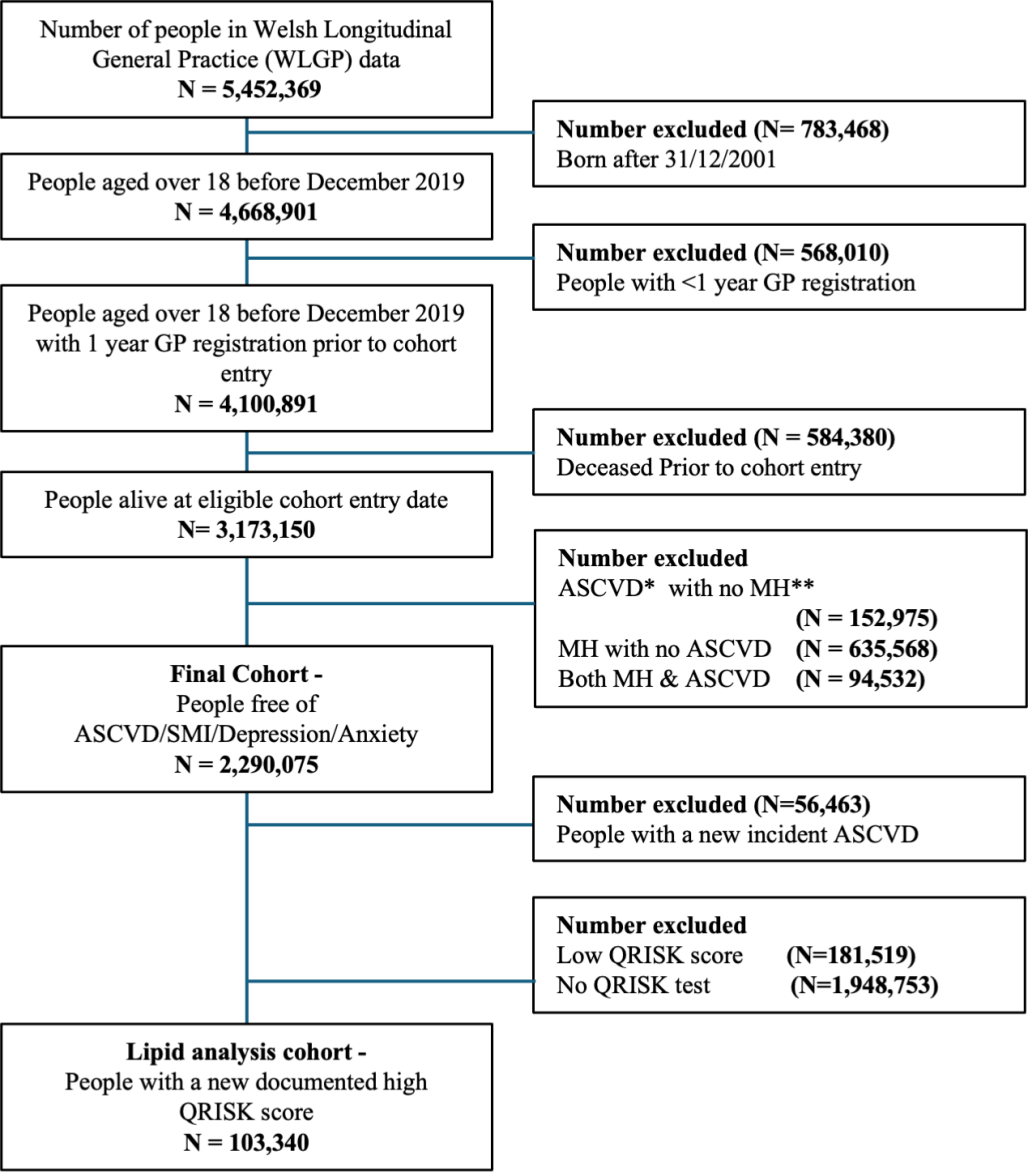
Flow chart showing cohort selection. ASCVD, atherosclerotic cardiovascular disease; GP, general practice data; MH, mental health conditions; SMI, severe mental illness.

Of the 284 859 (12.8% of the eligible population) patients with a documented QRISK score (total population 2 233 612), 103 340 (36.3%) had a first HQR documented, of whom 97 366 (94.2%) were non-depressed, 5204 (5.0%) depressed pre-HQR and 770 (0.8%) depressed post-HQR documentation (figure 1 and table 1).

**Table 1:** Patient characteristics at index date by depression status

	Non-depressed (n=97 366)	Depression pre-HQR (n=5204)	Depression post-HQR (n=770)	P value
Age, mean (SD**)**	65.2 (8.5)	59.8 (9.1)	61.6 (10.1)	<0.001
Sex				
Male	61 064 (62.7)	3130 (60.1)	438 (56.9)	
Female	36 302 (37.3)	2074 (39.9)	332 (43.1)	<0.001
Deprivation quintiles, WIMD				
1 (most deprived)	15 758 (16.2)	1432 (27.5)	169 (22.0)	
2	17 312 (17.8)	1115 (21.4)	174 (22.6)	
3	21 090 (21.7)	928 (17.8)	151 (19.6)	
4	20 431 (21.0)	846 (16.3)	149 (19.4)	
5 (least deprived)	21 290 (21.9)	825 (15.9)	115 (14.9)	<0.001
Location of residence				
Rural	34 688 (35.6)	1356 (26.1)	240 (31.2)	
Urban	61 768 (63.4)	3824 (73.5)	524 (68.1)	<0.001
Smoking status				
Current smoker	6874 (7.1)	789 (15.2)	100 (13.0)	
Ex smoker	34 991 (35.9)	1981 (38.1)	320 (41.6)	
Never smoked	55 501 (57.0)	2434 (46.8)	350 (45.5)	<0.001
Medical history				
Diabetes	7582 (7.8)	660 (12.7)	82 (10.7)	<0.001
Chronic kidney disease	284 (0.3)	18 (0.4)	<5 (NA)	0.55
Liver disease	1702 (1.8)	194 (3.7)	18 (2.3)	<0.001
Hypertension	44 765 (46.0)	2347 (45.1)	362 (47.0)	0.39

HQR, high QRISK score; WIMD, Welsh Index of Multiple Deprivation.

Of those with a HQR documented within the study period, both those with depression before and after first documentation of HQR were younger than non-depressed, comprised a higher proportion of females, were more deprived and were more likely to live in urban areas (table 1). These patients were also more likely to be current smokers or ex-smokers and more likely to have a history of diabetes mellitus or liver disease than non-depressed patients.

### LLT prescribing

Out of the 1 03 340 patients with HQR documentation, only 23 254 (22.5%) were prescribed LLT within 6 months of this date. Non-depressed patients were less likely to have LLT prescribed in this time frame compared with those with depression (22.3% non-depressed vs 26.2% depression pre-HQR and 29.9% depression post-HQR) (table 2). Among the whole population who were prescribed LLT, 8.0% were prescribed high-intensity statins, while 91.1% received low-intensity statins. However, non-depressed patients were slightly less likely to be prescribed high-intensity statins (7.9% non-depressed vs 10.0% depression pre-HQR and 11.4% depression post-HQR p<0.001).

**Table 2:** Number (%) of patients prescribed lipid lowering therapy within 6 months of a documented high QRISK score by sex, age, deprivation and location of residence

	Non-depressed	Depression pre-HQR	Depression post-HQR	P value	Within non-depressed	Within depression pre-HQR	Within depression post-HQR
					P value	P value	P value
Total	21 661 (22.3)	1363 (26.2)	230 (29.9)	<0.001			
Age group, years							
18–39	89 (29.2)	20 (29.4)	NA (NA)*	<0.001			
40–59	5225 (22.2)	635 (25.8)	101 (31.0)	<0.001			
60–74	13 411 (21.8)	631 (26.6)	104 (30.1)	<0.001			
75+	2936 (24.3)	77 (25.8)	NA (NA)*	<0.001	<0.001	<0.001	<0.001
Sex							
Male	13 749 (22.5)	821 (26.2)	119 (27.2)	<0.001			
Female	7912 (21.8)	542 (26.1)	111 (33.4)	<0.001	<0.001	<0.001	0.60
Deprivation quintiles, WIMD							
1 (most deprived)	3848 (24.4)	390 (27.2)	56 (33.1)	<0.001			
2	3989 (23.0)	309 (27.7)	49 (28.2)	<0.001			
3	4527 (21.5)	225 (24.3)	42 (27.8)	<0.001			
4	4334 (21.2)	204 (24.1)	45 (30.2)	<0.001			
5 (least deprived)	4642 (21.8)	222 (26.9)	35 (30.4)	<0.001	<0.001	<0.001	0.25
Location of residence							
Rural	6896 (19.9)	296 (21.8)	61 (25.4)	<0.001			
Urban	14 579 (23.6)	1061 (27.8)	167 (31.9)	<0.001	<0.001	<0.001	<0.001

* Governance restrictions within SAIL prohibit the reporting of numbers <5 due to privacy protection and disclosure control.

HQR, high QRISK score; WIMD, Welsh Index of Multiple Deprivation.

**Figure 2:**
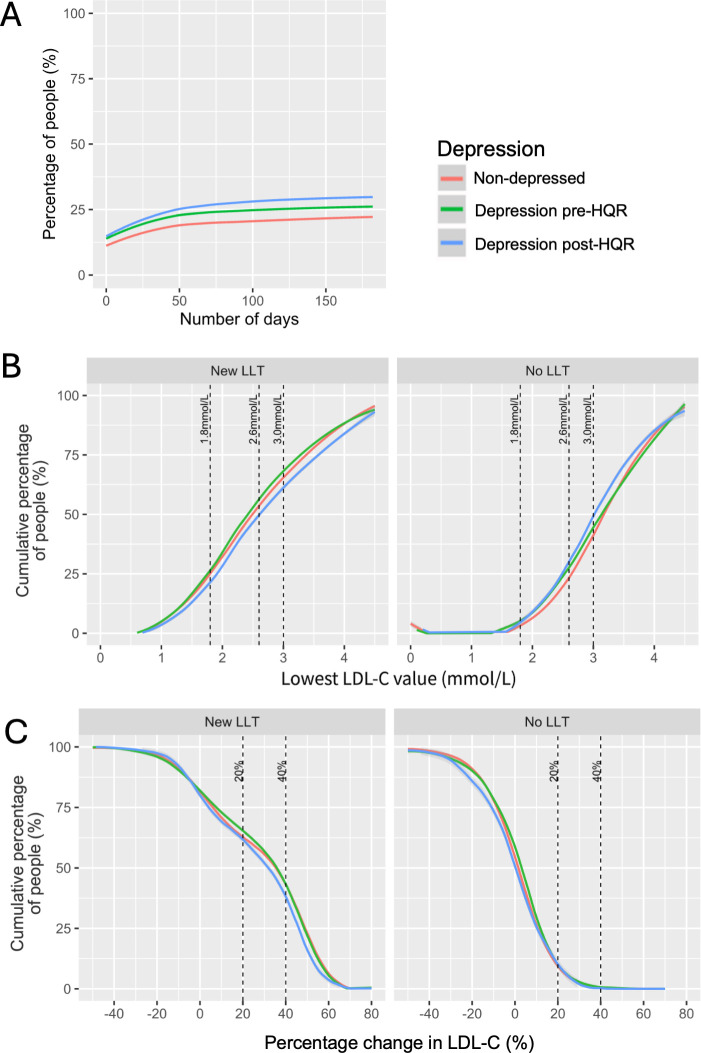
**(A**) Time in days from documented high QRISK score (HQR) to prescription of lipid lowering therapy by depression group. (**B**) Minimum low density lipoprotein level documented within 1 year of HQR in those with and without a prescription for lipid lowering therapy (number tested post HQR documentation=39 977 (38.7% of cohort), online supplemental table 1. (C) Percentage change in low density lipoprotein level within 1 year of a high QRISK score documentation in those with and without a prescription for lipid lowering therapy. LDL-C, low density lipoprotein cholesterol; LLT, lipid lowering therapy.

Patients in the depressed groups were more likely to be prescribed LLT on the day of HQR documentation than non-depressed. Patients with depression post-HQR were prescribed LLT earlier after HQR documentation than both non-depressed and depression pre-HQR patients. Rates of LLT prescription were similar between non-depressed and depression pre-HQR patients during the year post HQR documentation (figure 2A).

**Figure 3:**
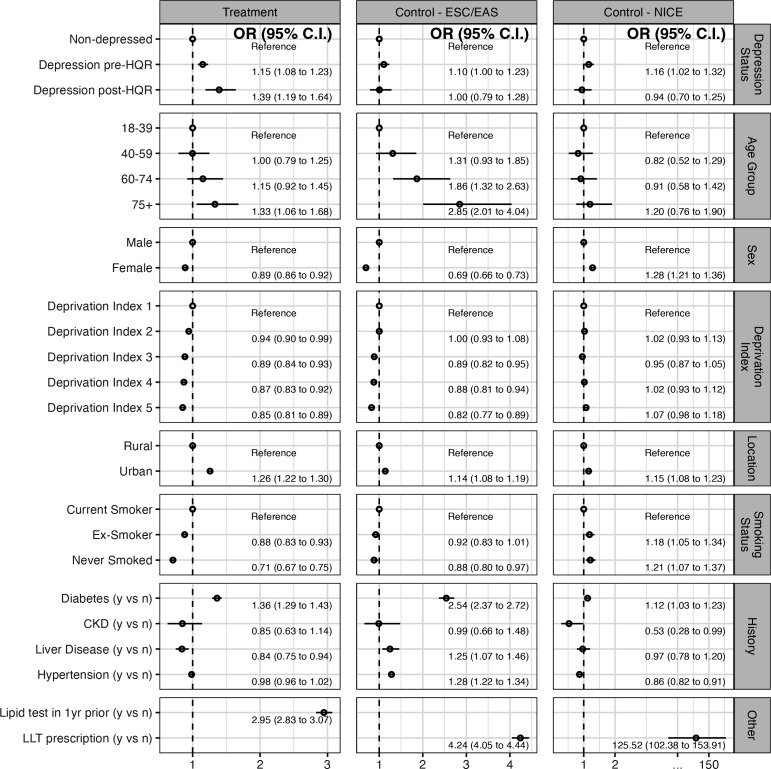
Multivariable analysis of (A) lipid lowering therapy prescription within 6 months of documented high QRISK score. (B) Achievement of ESC guideline target of low density lipoprotein cholesterol <2.6 mmol/L within 1 year of documented high QRISK score. (C) Achievement of NICE guidance of a >40% reduction in low density lipoprotein within 1 year of documented high QRISK score. CKD, chronic kidney disease; EAS, European Atherosclerosis Society; ESC, European Society of Cardiology, HQR, high QRISK score; LLT, lipid lowering therapy; NICE, National Institute for Health and Care Excellence.

Multivariable analysis (figure 3A) identified an association between depression status and receipt of LLT prescription, with the depression post-HQR group being the most likely to be prescribed LLT within 6 months of their HQR documentation, followed by the depression pre-HQR group. Females were less likely to be prescribed LLT than males, but age group was not associated with LLT prescription.

Being in the most deprived quintile, living in an urban location and current smoking were all associated with increased likelihood of receiving LLT. Prior lipid testing was a strong indicator of LLT prescription.

Interactions were explored between depression*age group, depression*sex, depression*deprivation and depression*location of residence but were not significant.

### LDL control: 2016 ESC/EAS lipid guideline target

Only 39 977 (38.7%) of the HQR cohort had a LDL-C test documented during the first year post-HQR documentation. HQR patients with LDL-C level documentation were more likely to have been prescribed LLT than those without documented levels (56.1% vs 43.9%, online supplemental table 1). A greater proportion of females were tested than males (40.5% vs 37.6%, online supplemental table 1).

A greater proportion of depression pre-HQR patients did not have a LDL-C test than those without depression, with depression post HQR least likely to have LDL-C testing (36.2% vs 38.8% vs 43.4%, respectively, p<0.001, online supplemental table 1).

In patients whose LDL-C was tested within 1 year of having a documented HQR score, those with depression were more likely to have a LDL-C level meeting the 2016 ESC/EAS ‘high risk’ primary prevention target of <2.6 mmol/L (39.8% depression post-HQR vs 39.3% depression pre-HQR and 36.9% non-depressed, <0.001 online supplemental table 2).

As expected, patients prescribed LLT were more likely to achieve guideline target LDL-C levels than those not on LLT (figure 2B). The proportion of non-depressed and depression pre-HQR achieving the ESC/EAS LDL-C target level was similar within those prescribed and not prescribed LLT.

Multivariable analysis showed that depression status was not associated with the documentation of a LDL-C <2.6 mmol/L within 1 year of a HQR documentation (figure 3B). Being prescribed LLT was the strongest predictor of achieving the LDL-C target. Females were less likely to achieve the guideline target than males, with likelihood of control rising with increasing deprivation (figure 3B).

Interactions were explored between depression and age group, sex, deprivation and location of residence but none were found to be significant.

### LDL control: National Institute for Health and Care Excellence (NICE-CG181 2014) guideline target

Of the 39 977 with a LDL-C recorded in the year post HQR documentation, 29 824 (74.6%) had a LDL-C test documented during the preceding year. A greater proportion of depressed patients (78.1%) had documented LDL-C levels both pre-HQR and post-HQR documentation than non-depressed (74.4%) (online supplemental table 1). Of those that had LDL-C recorded both pre-HQR and post-HQR documentation, only 7876 (26.4%) met the NICE guideline target of a >40% reduction in LDL-C from the pre-documentation to post-documentation LDL-C. Non-depressed patients were the least likely to achieve NICE LDL-C target, whereas those with depression post-HQR were the most likely although still only a low proportion (26.3% non-depressed compared with 27.8% depression pre-HQR and 29.5% depression post-HQR) (online supplemental table 3). The majority of patients that achieved the target had a LLT prescription (non-depressed 93.8%, depression pre-HQR 94.8% and depression post-HQR 92.1%) (figure 2C).

Female patients were more likely to achieve the guideline target than males in the non-depressed and depression pre-HQR groups (online supplemental table 3). For differences between other key characteristics see online supplemental table 3.

Multivariable analysis showed that depression pre-HQR patients were more likely to achieve the NICE guideline target >40% reduction in LDL-C, but no association was found with depression post-HQR (figure 3C). Females were more likely to meet the guideline target than males. Prescription of LLT was the strongest predictor in achieving the target.

Interactions were explored between depression and age group, sex, deprivation and location of residence but none were found to be significant.

Online supplemental figure 1 illustrates achievement of both the NICE and ESC/EAS guideline targets. The majority of patients, independent of depression status, did not achieve either guideline target (LDL-C ≥2.6 mmol/L and <40% reduction).

## Discussion

This study investigated differences in LLT prescribing and achievement of guideline target levels in patients with and without depression after first documentation of being categorised as at high risk of ASCVD according to UK guideline recommended QRISK score criteria. Overall, QRISK score was sparsely documented and when HQR was recorded fewer than one in four of those patients were prescribed LLT within 6 months. Those with depression (whether identified pre-HQR or post-HQR documentation) were more likely to be prescribed LLT than those without. Lipid testing rates were very low within the first year post HQR documentation with a low proportion of patients achieving the ESC/EAS guideline target of <2.6 mmol/L, but similar in those with and without depression. However, those with depression pre-HQR documentation were more likely to achieve the NICE guideline reduction of >40% in those with documented LDL-C levels.

The overall low documentation of QRISK scores irrespective of depression status suggests that assessment of cardiovascular risk is a low clinical priority in routine care of the general population free from ASCVD in Wales. This could mean that a significant proportion of patients who, if tested, would be classified as at high ASCVD remain unidentified and are not benefiting from early primary prevention management. Somewhat concerningly, despite greater prescription of LLT, patients with depression were no more likely to achieve ESC/EAS guidelines than those without depression. This finding did differ to that of achievement of the NICE guideline target where patients with depression pre-HQR documentation were more likely to achieve the >40% reduction in LDL-C than those with post-HQR depression and without depression. This discrepancy may be due to NICE recommending a percentage reduction in LDL-C with some patients achieving this but remaining above the ESC/EAS target of <2.6 mmol/L. Concerningly, it was only possible to undertake an analysis of NICE target achievement in the very small population for whom there were lipid test results both pre-HQR and post-HQR documentation, which may reflect a group with better overall patient engagement and care.

The more frequent prescribing of LLT documented in those with depression agrees with previous work by Garriga *et al* in a primary prevention population.^24^ Others have shown that patients with depression were less likely to achieve guideline LDL-C targets in both the primary and secondary ASCVD prevention setting.^3 4^ While our study focused on potential differences in care between those with and without depression, it should be noted that overall LLT prescribing in those with a guideline-recommended indication for LLT was very low (less than a quarter of those with documented HQR), with fewer patients prescribed LLT than not, despite evidence-based guideline-endorsed recommendations for LLT in these patients irrespective of depression status.

There may be a number of reasons underpinning why greater LLT prescribing did not translate into improved ESC/EAS guidance levels of lipid level control in the depressed patients. This could include lack of adherence to the LLT as well as other behavioural and physiological factors associated with dyslipidaemia in those with depression such as inferior dietary quality and/or lower physical activity.^425^,^27^ However, it is not possible to identify all the reasons for this in a study of this design, without direct access to the patients and practitioners. Further studies would be needed to investigate the above issues.

Unsurprisingly, patients prescribed LLT were much more likely to achieve LDL-C targets than those not receiving such therapy. However, only a very low proportion of patients had an LDL-C check within 1 year of documented HQR. Thus, only a very small proportion of those patients at high risk of ASCVD events were documented as having achieved LDL-C targets within the first year after their high-risk status was entered in their EHR. Such low rates of testing and follow-up in this high-risk population are consistent with previous wider-ranging studies and not unique to Wales.^28^,^30^ Therefore, our findings highlight a universal need for improved strategies to target the many patients not currently receiving appropriate risk stratification, subsequent initiation of LLT and appropriate follow-up monitoring where indicated. It is noteworthy that the removal of financial incentives for GPs in Wales regarding risk factor management in 2014–2015, including lipids (recording of total cholesterol ≤5 mmol/L within the previous 15 months in coronary heart disease and diabetic patients), coincided with a steeper decline in lipid testing, treatment and target achievement. This raises the question of whether reinstatement of an incentive structure may lead to better management and clinical outcomes.^31 32^

### Limitations

As with many studies using routinely collected observational data, the information was not originally gathered for research purposes. Therefore, there may be some inaccuracies within the data both entered and other relevant data that have not been entered. It is not possible to investigate the impact of adherence to LLT on control of lipid levels as dispensing and pill consumption data are not available within the SAIL Databank.

This study focused on lipid assessment and management for primary prevention within the primary care environment, where most patients are managed in Wales and thus this analysis is a fair reflection of care for the vast majority of such patients within Wales. These findings should be generalisable to areas with similar healthcare systems and other countries that manage ASCVD prevention within the primary care environment, although some differences may be seen comparing systems that fund preventative medications differently to the UK.

PCSK9 inhibitor prescriptions have not been included within the list of LLT. These were not approved for use within the National Health Service until 2016.^33 34^ These agents are prescribed through specialist hospital outpatient clinics, for which the data are not available, and still are only infrequently prescribed due to relatively restrictive NICE guidance. It is therefore unlikely that inclusion of this small number of patients would have materially impacted our study outcomes.^34^

While a broad definition of depression was used in this study, routinely collected EHR data do not allow for easy distinction of differences in the severity, duration and recurrence of depression in these patients (particularly between patients categorised by a depression diagnosis code vs those with depressive symptoms plus antidepressants), nor the clustering of depressive symptoms that may favour the development of ASCVD. There may therefore be a subgroup of depressed patients not identified within this study who have a greater risk of ASCVD and who would benefit from further investigation. Similarly, it is difficult to know when patients first developed depression, thus the documentation of a diagnosis can only act as a marker. Therefore, patients in the post-HQR group may have had depression pre-documentation of HQR or post-LLT prescription. However, in this case, depression diagnosis was considered as a *‘signpost*’ for determining primary prevention management, rather than to investigate any potential influence on the development of atherosclerotic risk. It is also important to note that due to the nature of the database and inclusion criterion of a documented HQR, only those patients who have engaged with their GP, whether depressed or not, were available for inclusion in these analyses. Future work looking more prospectively at individual patient utilisation of preventive services and cardiovascular outcomes may help better understand the impact of discrepancies in quality of care.

As noted previously, there was very low documentation of any QRISK cardiovascular risk scores in the EHRs; there may be patients who had a score calculated and discussed in clinic but not recorded and as such are unknown. This may in part reflect clinician behaviour and could confound the associations with prescribing and control. QRISK scores were not imputed ‘post-hoc’, as we wished to examine effectiveness of lipid management in routine care specifically in those with a risk score recorded. Therefore, it is only possible to assess LLT prescribing and lipid level control in the small proportion of patients whose record contains a documented QRISK score above the ‘high-risk’ threshold. While LLT prescribing data are available within the SAIL Databank, it is not possible to access the free text within the GP records, which may provide additional information about whether patients have refused statin therapy or initially selected lifestyle interventions for reducing lipid levels (despite recommendation for statin therapy in the guidelines). It was not possible to look at the impact of lipid management on future CV events within the constraints of this study; however, it would be expected that the lower frequency of testing, treatment and control of lipids could lead to worse outcomes in the population. Indeed, Yeh *et al* found that patients with symptomatic ASCVD with uncontrolled LDL-C were more likely to have a major adverse CV event.^35^

## Conclusion

In conclusion, while patients with depression were more likely to be prescribed LLT this was not necessarily reflected in the level of control of subsequent lipid levels, which was similar to non-depressed patients. In addition, the low levels of QRISK documentation, lipid testing, LLT prescribing and control of lipid levels in all patients independent of depression status need addressing.

## Supplementary material

10.1136/openhrt-2025-003800 online supplemental file 1

## Data Availability

Data may be obtained from a third party and are not publicly available.
