# Supplementary material for: Treatment and control of low-density lipoprotein for primary prevention in patients in Wales with and without depression: a study of whole-population electronic health records
Source: Open Heart. 2026 Jun 22;13(1):e003800. doi: 10.1136/openhrt-2025-003800 (PMC13289360; doi:10.1136/openhrt-2025-003800)
Supplement: online supplemental file 1 [file openhrt-13-1-s001.docx]

**SUPPLEMENT**


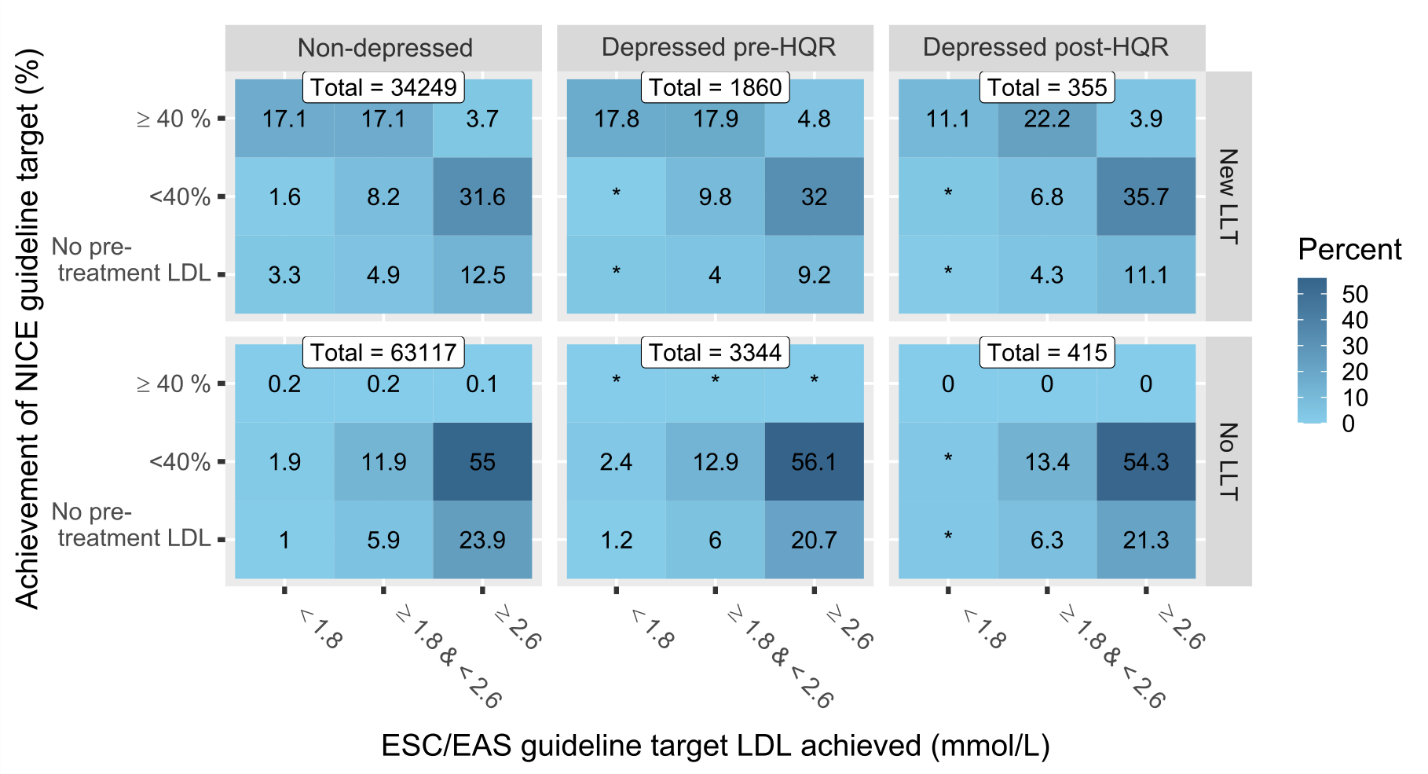


Supplemental Figure 1: Grid plot showing achievement of ESC/EAS and NICE guideline targets in patients within 1 year of documentation of a high QRISK score by depression and lipid lowering therapy prescription status. * Governance restrictions within SAIL prohibit the reporting of numbers <5 due to privacy protection and disclosure control.

Supplemental Table 1: Number of people with and without a low density lipoprotein test recorded within 1 year of documentation of a high QRISK score.

|  | **LDL-C test** | **No test** | **p** |
| --- | --- | --- | --- |
| **Depression** |  |  |  |
| Non-depressed | 37757 (38.8) | 59609 (61.2) |  |
| Depression pre-high QRISK score | 1886 (36.2) | 3318 (63.8) |  |
| Depression post-high QRISK score | 334 (43.4) | 436 (56.6) | < 0.001 |
| **Age group (years)** |  |  |  |
| 18-39 | 165 (42.5) | 223 (57.5) |  |
| 40-59 | 9755 (37.0) | 16593 (63.0) |  |
| 60-74 | 25336 (39.5) | 38803 (60.5) |  |
| 75+ | 4721 (37.9) | 7744 (62.1) | < 0.001 |
| **Lipid lowering therapy** |  |  |  |
| No LLT | 19507 (29.2) | 47369 (70.8) |  |
| New LLT | 20470 (56.1) | 15994 (43.9) | < 0.001 |
| **Sex** |  |  |  |
| Male | 24304 (37.6) | 40328 (62.4) |  |
| Female | 15673 (40.5) | 23035 (59.5) | < 0.001 |
| **Deprivation quintile WIMD** |  |  |  |
| 1 (most deprived) | 6740 (38.8) | 10619 (61.2) |  |
| 2 | 7125 (38.3) | 11476 (61.7) |  |
| 3 | 8697 (39.2) | 13472 (60.8) |  |
| 4 | 8025 (37.5) | 13401 (62.6) |  |
| 5 (least deprived) | 8816 (39.7) | 13414 (60.4) | < 0.001 |
| **Location of residence** |  |  |  |
| Rural | 13809 (38.1) | 22475 (61.9) |  |
| Urban | 25824 (39.1) | 40292 (60.9) | 0.003 |
| **Tested pre & post-high QRISK score** |  |  |  |
| Non-depressed | 28091 (74.4) | 9666 (25.6) |  |
| Depression pre-high QRISK score | 1473 (78.1) | 413 (21.9) |  |
| Depression post-high QRISK score | 261 (78.1) | 73 (21.9) | <0.001 |

Supplementa1 Table 2: Number (%) of patients with and without depression with a documented LDL-C at the ESC/EAS 2016 guideline level of <2.6mmol/L, within 1 year of a documented high QRISK score by sex, age, deprivation and location of residence.

|  |  |  |  |  | | **Within non-depressed** | | **Within depression pre-HQR** | | **Within depression post-HQR** | |
| --- | --- | --- | --- | --- | --- | --- | --- | --- | --- | --- | --- |
|  | **Non-depressed** | **Depression pre-HQR** | **Depression post-HQR** | **p** | | **p** | | **p** | | **p** | |
| **Total** | 13933 (36.9) | 741 (39.3) | 133 (39.8) | < 0.001 | |  | |  | |  | |
| **Age group (years)** |  |  |  |  | |  | |  | |  | |
| 18-39 | 51 (38.1) | 10 (37.0) | NA^*^ | <0.001 | |  | |  | |  | |
| 40-59 | 2998 (34.2) | 324 (38.0) | 57 (39.3) | <0.001 | |  | |  | |  | |
| 60-74 | 8877 (36.6) | 358 (39.7) | 56 (37.8) | <0.001 | |  | |  | |  | |
| 75+ | 2007 (43.8) | 49 (46.7) | NA^*^ | <0.001 | | <0.001 | | <0.001 | | <0.001 | |
| **Sex** |  |  |  |  | |  | |  | |  | |
| Male | 9009 (39.1) | 450 (40.7) | 71 (40.3) | <0.001 | |  | |  | |  | |
| Female | 4924 (33.4) | 291 (37.3) | 62 (39.2) | <0.001 | | <0.001 | | <0.001 | | 0.44 | |
| **Deprivation quintiles WIMD** |  |  |  |  | |  | |  | |  | |
| 1 (most deprived) | 2492 (40.5) | 183 (35.5) | 25 (39.7) | <0.001 | |  | |  | |  | |
| 2 | 2597 (39.2) | 179 (41.5) | 31 (40.8) | <0.001 | |  | |  | |  | |
| 3 | 3022 (36.3) | 115 (37.3) | 21 (32.3) | <0.001 | |  | |  | |  | |
| 4 | 2710 (35.5) | 126 (41.6) | 33 (42.3) | <0.001 | |  | |  | |  | |
| 5 (least deprived) | 2922 (34.6) | 134 (42.7) | 18 (40.9) | <0.001 | | <0.001 | | <0.001 | | 0.17 | |
| **Location of residence** |  | | | |  | |  | |  | |  |
| Location - Rural | 4548 (34.4) | 167 (33.7) | 39 (37.1) | <0.001 | |  | |  | |  | |
| Location - Urban | 9276 (38.3) | 571 (41.3) | 92 (40. 9) | <0.001 | | <0.001 | | <0.001 | | <0.001 | |

* Governance restrictions within SAIL prohibit the reporting of numbers <5 due to privacy protection and disclosure control. HQR high QRISK

Supplementa1 Table 3: Number (%) of patients with and without depression that achieved the recommended NICE guideline of a >40% reduction in low density lipoprotein within 1 year of high QRISK score documentation by sex, age, deprivation and location of residence.

|  |  |  |  |  | **Within non-depressed** | **Within depression pre-HQR** | **Within depression post-HQR** |
| --- | --- | --- | --- | --- | --- | --- | --- |
|  | **Non-depressed** | **Depression pre-HQR** | **Depression post-HQR** | **p** | **p** | **p** | **p** |
| **Total** | 7390 (26.3) | 409 (27.8) | 77 (29.5) | <0.001 |  |  |  |
| **Age group (years)** |  |  |  |  |  |  |  |
| 18-39 | 27 (25.2) | 5 (20.8) | NA^*^ | <0.001 |  |  |  |
| 40-59 | 1510 (22.9) | 177 (26.8) | 36 (28.8) | <0.001 |  |  |  |
| 60-74 | 4801 (26.8) | 209 (29.2) | 31 (29.3) | <0.001 |  |  |  |
| 75+ | 1052 (30.0) | 18 (24.3) | NA^*^ | <0.001 | <0.001 | <0.001 | <0.001 |
| **Sex** |  |  |  |  |  |  |  |
| Male | 4266 (25.7) | 227 (26.6) | 35 (27.0) | <0.001 |  |  |  |
| Female | 3124 (27.2) | 182 (29.5) | 42 (32.1) | <0.001 | <0.001 | 0.026 | 0.43 |
| **Deprivation quintiles WIMD** |  |  |  |  |  |  |  |
| 1 (most deprived) | 1264 (27.1) | 102 (27.0) | 19 (38) | <0.001 |  |  |  |
| 2 | 1322 (27.2) | 95 (27.9) | 20 (31.3) | <0.001 |  |  |  |
| 3 | 1533 (24.8) | 68 (27.9) | 11 (22.0) | <0.001 |  |  |  |
| 4 | 1459 (26.0) | 56 (23.1) | 15 (26.3) | <0.001 |  |  |  |
| 5 | 1708 (26.8) | 87 (35.1) | 9 (26.5) | <0.001 | <0.001 | 0.001 | 0.18 |
| **Location of residence** |  |  |  |  |  |  |  |
| Rural | 2306 (23.7) | 85 (22.0) | 17 (22.4) | <0.001 |  |  |  |
| Urban | 5026 (27.8) | 324 (30.1) | 58 (32.0) | <0.001 | <0.001 | <0.001 | <0.001 |

* Governance restrictions within SAIL prohibit the reporting of numbers <5 due to privacy protection and disclosure control. HQR high QRISK
